# Prevalence, treatment patterns, and healthcare resource utilization in Medicare and commercially insured non-dialysis-dependent chronic kidney disease patients with and without anemia in the United States

**DOI:** 10.1186/s12882-018-0861-1

**Published:** 2018-03-15

**Authors:** Wendy L. St. Peter, Haifeng Guo, Shaum Kabadi, David T. Gilbertson, Yi Peng, Trudy Pendergraft, Suying Li

**Affiliations:** 1 Chronic Disease Research Group, Minneapolis Medical Research Foundation, 701 Park Avenue, Suite S4.100, Minneapolis, MN 55455 USA; 2 College of Pharmacy, University of Minnesota, Minneapolis, MN USA; 3 AstraZeneca, Wilmington, Delaware, USA

**Keywords:** Anemia, Anemia treatment, Healthcare utilization, Non-dialysis-dependent chronic kidney disease

## Abstract

**Background:**

Anemia is common in non-dialysis-dependent chronic kidney disease (NDD-CKD) patients, but detailed information on prevalence and treatment is lacking.

**Methods:**

We evaluated anemia prevalence and treatment using two datasets: the Medicare 20% random sample (ages 66–85 years), and the Truven Health MarketScan database (ages 18–63 years). We selected stage 3–5 NDD-CKD patients with and without anemia from both databases during 2011–2013. We evaluated anemia prevalence and treatment (erythropoietin stimulating agents [ESAs], intravenous [IV] iron, red blood cell [RBC] transfusions) following anemia diagnosis during a 1-year baseline period, and healthcare utilization during a 1-year follow-up period. We used Poisson regression models to compare healthcare utilization in patients with and without anemia, adjusting for demographics, baseline comorbid conditions, inflammatory conditions, and CKD stage.

**Results:**

We identified 218,079 older and 56,188 younger stage 3–5 NDD-CKD patients. Anemia prevalence increased with age in both datasets; was higher in women, black patients (Medicare only), and patients with comorbid conditions; and rose sharply with increasing CKD stage. Of 15,716 younger anemic patients, 11.7%, 10.8%, and 9.4% were treated with RBC transfusion, ESAs, and IV iron, respectively. Corresponding proportions of 109,251 older anemic patients were 22.2%, 12.7%, and 6.7%. Regardless of age, anemic patients were more likely than non-anemic patients to use healthcare resources, including hospitalizations and emergency department, hematologist, nephrologist, and outpatient visits. Anemic NDD-CKD patients were more likely to be treated with RBC transfusion than with ESAs or IV iron.

**Conclusion:**

More research is necessary to determine best approaches to anemia management in CKD.

**Electronic supplementary material:**

The online version of this article (10.1186/s12882-018-0861-1) contains supplementary material, which is available to authorized users.

## Background

Anemia is common in patients with non-dialysis-dependent (NDD) chronic kidney disease (CKD), and prevalence increases as CKD worsens [1]. The most current data on anemia prevalence in NDD-CKD patients are from the 2007–2010 National Health and Nutrition Examination Survey (NHANES) [2]. These data are representative of the United States population, but the sample size is relatively small and does not allow for subgroup analysis. Thus, more recent anemia prevalence information using a larger dataset is necessary to allow evaluation of trends by key factors such as age, sex, and race. Much attention has been focused on anemia treatment in patients with stage 5 CKD on dialysis. However, relatively little is known about anemia treatment patterns in NDD-CKD stage 3–5 patients. Thamer and colleagues retrospectively evaluated erythropoiesis-stimulating agent (ESA) therapy in commercially insured NDD-CKD patients before and after publication of TREAT (Trial to Reduce Cardiovascular Events with Aranesp Therapy) results in 2009 [3]. They showed that, post-TREAT, about 8% of CKD patients were prescribed ESAs by September 2011, with higher prescription rates in stage 4 than in stage 3 patients. They did not assess treatment patterns for iron therapy or red blood cell (RBC) transfusion use.

TREAT results showed that targeting anemia treatment to a hemoglobin level of 13 g/dL with darbepoetin vs. placebo (rescue darbepoetin if hemoglobin < 9 g/dL) in NDD-CKD patients with diabetes did not reduce risk of death or cardiovascular events, but was associated with increased stroke risk [4]. Subsequent to TREAT, and with evidence from other randomized trials evaluating ESA treatment in NDD-CKD patients [5, 6], the US Food and Drug Administration (FDA) revised ESA product labels in June 2011, adding new boxed warnings and recommending more conservative dosing [7]. In 2012, the Kidney Disease Improving Global Outcomes (KDIGO) guideline group released updated practice guidelines for anemia in CKD [8]. The FDA action and KDIGO guidelines were intended to influence anemia treatment among NDD-CKD patients.

Anemia has been identified as a risk factor for increased hospitalizations and/or hospital days in NDD-CKD patients [9, 10], but more research is needed regarding how anemia may affect other healthcare utilization, such as emergency department (ED) or clinic visits or specialty care.

Our study objectives were to: 1) update anemia prevalence estimates in Medicare-covered and commercially insured stage 3–5 NDD-CKD patients; 2) examine anemia prevalence in these patients by CKD stage, age, sex, and race; 3) evaluate treatment patterns in NDD-CKD patients in a contemporary period after TREAT and the enhanced FDA boxed warning, and around the time of the updated KDIGO anemia guidelines release; and 4) assess healthcare burden/utilization in NDD-CKD patients with and without anemia.

## Methods

### Study population and data sources

We evaluated two study populations: patients aged 66–85 years from the 20% Medicare random sample, and younger commercially insured patients aged 18–63 years from the Truven Health MarketScan database. Medicare data include enrollment information, demographic characteristics, and medical claims from Part A, Part B, and Part D. The Medicare claims files contain information collected by Medicare to allow payment for healthcare services provided to Medicare beneficiaries in the US and its territories. Standard analytic files (SAFs) generated by the Centers for Medicare & Medicaid Services (CMS) were used. SAFs are available for each institutional claim type (inpatient, outpatient, skilled nursing facility, home health agency, carrier, or hospice). Non-institutional Part B physician/supplier SAFs include final action claims for physician services, laboratory services, and durable medical equipment. CMS Part D data include the prescription drug event file, which contains the National Drug Index code (NDC) for each drug, prescription dosing information, and drug costs.

The Truven Health MarketScan Commercial Claims and Encounters Database includes specific health services records for employees and their dependents from a selection of large employers, health plans, and government and public organizations. Information from various MarketScan tables can be merged using a unique patient identifier provided in the data. Similar to the Medicare Part D data, prescription drug information is available in the MarketScan database [11].

### Study sample and patient selection

We selected stage 3–5 NDD-CKD patients with and without anemia from the 20% Medicare sample and MarketScan databases for the study period 2011–2013. Patients in the 20% Medicare data were included if they met the following criteria: (1) age at least 66 years as of the end date of a 1-year baseline period; (2) Medicare both Parts A and B coverage; 3) not in a health maintenance organization; 4) not on dialysis during the baseline period or on the first day of the follow-up period; and (5) alive on the first day of the follow-up period. Patients in the commercially insured population were included if they met the following criteria: (1) age 18–63 years as of the end date of a 1-year baseline period; (2) commercial insurance coverage; (3) not on dialysis during the baseline period; and (4) enrolled in the commercial insurance claim dataset for at least 1 day during a follow-up period. Patients in both data sources with end-stage renal disease (ESRD) in the baseline period were excluded.

### Study design

**Fig. 1:**
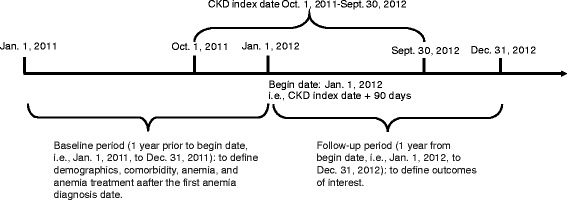
Study design for 2012 cohorts of Medicare-covered and commercially insured (MarketScan) stage 3–5 non-dialysis-dependent chronic kidney disease patients. CKD, chronic kidney disease

This was a retrospective observational database study. We identified patients with a CKD index date from October 1, 2011, to September 30, 2012. As shown in Fig. 1, the baseline period was 1 year before the index date + 90 days, including the index date + 90 days; 90 days were added to the CKD index date to allow more time to ascertain anemia diagnoses and start of anemia treatment when anemia was diagnosed after CKD. We defined patient demographic characteristics, comorbid conditions, anemia, and anemia treatment during the baseline period. Follow-up was 1 year from the index date + 91 days; patients were censored at death (20% Medicare data only), end of Part A or B coverage (Medicare patients) or commercial insurance coverage (MarketScan patients), ESRD date, 365 days, or December 31, 2013. Healthcare utilization was defined during the follow-up period.

### CKD and anemia definitions

Patients with CKD were identified using *International Classification of Diseases, Ninth Revision, Clinical Modification* (ICD-9-CM) diagnosis codes (Additional file 1: Table S1**)** on one or more inpatient claims or two or more outpatient claims on different dates within 365 days. The CKD date was defined by the discharge date of an inpatient claim or the second outpatient date. Among CKD patients, if at least one claim carried a CKD stage code (585.1–585.5) during the same period that defined CKD, the code for the highest stage was used. In MarketScan data, if lab data were available, CKD stage was also defined using estimated glomerular filtration rate (eGFR) by the CKD-EPI 2009 equation [12].

We defined anemia during the baseline period using ICD-9-CM diagnosis codes on one or more inpatient claims, or two or more outpatient claims on different dates. We started with a broad definition including ICD-9-CM diagnosis codes 280–285. The final diagnosis codes were based on clinical relevance and distribution of each code and included iron and other deficiency anemia, acute posthemorrhagic anemia, anemia in CKD, anemia of other chronic disease, and other unspecified anemia (Additional file 1: Table S1).

### Anemia treatment

We assessed anemia treatment (ESAs, intravenous [IV] iron, and whole blood or packed RBC transfusions) immediately following anemia diagnosis during the baseline period, defining use by at least one claim for each treatment. When determining ESA use, we considered administration in outpatient clinics (identified using J codes and revenue center codes) and home use (identified using NDC codes from prescription drug data, including Medicare Part D). We also defined consistency of ESA treatment. Consistent use was defined as at least one ESA administration in at least 80% of available treatment months, and inconsistent use as at least one administration in less than 80% of available treatment months (see Additional file 1: Table S1 for definition and claim source details).

### Covariates

Comorbid and inflammatory conditions were defined as present if at least one inpatient claim or at least two outpatient or physician/supplier claims on different days were identified during the baseline period (see Additional file 1: Table S1 for specific diagnosis codes).

### Health outcomes

Healthcare utilization in the follow-up period was identified for all-cause hospitalizations, ED visits, skilled nursing facility (SNF) admissions, hospice admissions, outpatient clinic visits, and visits to nephrologist, cardiologist, and primary care practitioners, including general practice, family practice, internal medicine, geriatric medicine, nurse practitioner, and physician assistant.

### Statistical analysis

Anemia prevalence was calculated as the percentage of patients and reported overall and by CKD stages stratified by demographic characteristics and comorbid conditions. Distributions of age, sex, race (Medicare only), and presence of comorbidity are reported as percentages overall and by CKD stage. Anemia treatment rates were measured as percentages of anemia patients who received each type of treatment or combination. Healthcare utilization was expressed as number of claims per person-year during follow-up for stage 3–5 NDD-CKD patients, with and without anemia, overall and by CKD stage. We calculated the standardized difference for each baseline variable to compare differences in patient demographic characteristics and comorbidity burden in patients with and without anemia. Differences were considered significant if the absolute value of the standardized difference was greater than 10.

We compared healthcare utilization for patients with and without anemia. First, we calculated unadjusted healthcare utilization rates expressed as number of claims per patient-year, mean (median) number of hospitalization days, and number of physician visits per patient-year during the follow-up period. Considering that differences in healthcare utilization rates may be associated with the burden of comorbid conditions in anemia patients, we used multivariate Poisson regression models with number of each type of health service as the dependent variable. In each model, we further adjusted for demographic characteristics (age, sex, and race for Medicare only), baseline comorbid conditions (cardiovascular disease, diabetes, hypertension, liver disease, gastrointestinal bleeding, and cancer), and inflammatory conditions (chronic infections, Crohn’s disease, hepatitis C, and others), and CKD stage.

## Results

We identified 218,079 stage 3–5 NDD-CKD patients aged 66–85 years from the 20% Medicare sample and 56,188 patients aged 18–63 years from the commercially insured population during the study period (Additional file 2: Figure S1).

### Prevalence of anemia in stage 3–5 NDD-CKD patients

For younger patients (commercially insured), overall anemia prevalence was 28.0% (Table 1); prevalence increased with CKD progression (22.4%, stage 3; 41.3%, stage 4; 53.9%, stage 5). For older patients (Medicare), overall anemia prevalence was 50.1%; prevalence was 43.9%, 64.0%, and 72.8% for stages 3, 4, and 5, respectively. Prevalence increased with age in both data sets, and was higher in women and black patients (Medicare only) and much higher in patients with comorbid conditions except diabetes and hypertension.

**Table 1:** Prevalence of anemia in stage 3–5 NDD-CKD Patients in 20% Medicare and commercially insured (MarketScan) datasets

	Medicare					Commercially insured (MarketScan)				
		Anemia prevalence (%), CKD stage					Anemia prevalence (%), CKD stage			
Characteristics	*n,* anemia^a^	3–5	3	4	5	*n,* anemia^a^	3–5	3	4	5
Overall	109,251	50.1	43.9	64.0	72.8	15,716	28.0	22.4	41.3	53.9
Age, years										
18–44	─ ─	─ ─	─ ─	─ ─	─ ─	1724	25.9	19.6	34.9	50.4
45–54	─ ─	─ ─	─ ─	─ ─	─ ─	3755	27.0	21.4	40.1	50.6
55–59	─ ─	─ ─	─ ─	─ ─	─ ─	4450	28.5	23.0	41.5	56.4
60–63	─ ─	─ ─	─ ─	─ ─	─ ─	5787	28.9	23.3	44.2	56.8
66–69	15,500	44.6	38.0	59.9	69.0	─ ─	─ ─	─ ─	─ ─	─ ─
70–74	25,212	47.0	40.7	62.5	70.9	─ ─	─ ─	─ ─	─ ─	─ ─
75–79	29,625	50.1	44.2	63.6	72.9	─ ─	─ ─	─ ─	─ ─	─ ─
80–85	38,914	55.1	49.2	67.0	76.5	─ ─	─ ─	─ ─	─ ─	─ ─
Sex										
Men	50,615	46.9	41.1	60.3	69.5	7531	23.7	18.5	35.1	50.5
Women	58,636	53.2	46.7	67.4	76.2	8185	33.6	27.5	48.5	58.3
Race^b^										
White	87,617	48.9	42.9	62.9	71.9	─ ─	─ ─	─ ─	─ ─	─ ─
Black	15,263	57.9	51.0	70.5	75.2	─ ─	─ ─	─ ─	─ ─	─ ─
Other	6371	51.4	43.5	66.4	75.1	─ ─	─ ─	─ ─	─ ─	─ ─
Comorbidity										
ASHD	57,070	59.0	53.0	70.0	78.4	3707	39.7	33.0	51.3	65.3
CHF	44,239	66.9	61.1	74.9	82.4	3360	47.9	40.3	57.8	69.1
CVA/TIA	25,814	62.6	56.9	73.1	79.8	1573	44.9	38.3	55.3	67.6
PVD	38,868	62.8	57.0	72.2	80.6	2997	49.6	42.6	60.7	69.5
Cardiac (other)	38,822	65.6	59.7	76.1	82.7	3688	48.5	41.5	60.7	67.9
COPD	36,910	62.4	56.6	73.1	79.5	2467	43.5	37.6	54.1	67.2
GI bleeding	13,773	87.1	83.9	92.4	93.3	1403	71.7	66.2	83.0	79.0
Liver disease	4088	71.6	65.6	81.9	85.4	1118	52.2	44.9	60.5	70.3
Dysrhythmia	47,814	61.1	55.3	71.3	79.7	3060	44.4	37.4	56.4	67.6
Cancer	22,524	60.3	54.9	72.1	79.7	2227	41.7	35.3	57.8	69.2
Diabetes	64,180	54.0	47.3	66.7	76.2	8246	33.7	27.4	46.4	59.1
Hypertension	105,397	51.0	44.8	64.7	73.9	13,418	31.0	24.9	44.2	57.6
Inflammatory conditions										
Glomerulonephritis	6529	68.7	58.1	77.9	88.1	1229	44.1	34.7	52.8	75.0
Chronic infections	2128	74.7	70.4	78.8	89.3	622	56.2	48.7	70.7	72.4
Crohn’s disease	836	70.5	64.2	80.4	90.3	199	50.3	47.2	53.4	65.9
Ulcerative colitis	912	71.4	66.4	82.4	87.7	176	55.0	48.9	62.0	83.8
Hepatitis C	703	66.7	61.5	72.2	77.0	354	48.2	42.8	50.4	62.7
Gout	17,862	59.0	50.8	70.5	80.8	1521	32.2	26.0	43.8	59.6
Rheumatoid arthritis	5593	67.4	61.8	78.8	87.6	495	39.8	35.9	55.6	53.9

*ASHD* atherosclerotic heart disease, *CHF* congestive/chronic heart failure, *CKD* chronic kidney disease, *COPD* chronic obstructive pulmonary disease, *CVA/TIA* cerebrovascular accident/transient ischemic attack, *GI* gastrointestinal, *NDD* non-dialysis-dependent, *PVD* peripheral vascular disease

^a^In the row “Overall,” *n* is the total number of patients with anemia. In other rows, *n* represents the number of anemia patients in each subgroup; for example, the *n* of 57,070 in the row “ASHD” represents the number of anemia patients with comorbid ASHD

^b^Race variable is not available in the MarketScan database

### Patient characteristics in stage 3–5 NDD-CKD patients with and without anemia

In both data sets, prevalence of comorbid and inflammatory conditions was generally higher in patients with than without anemia (Additional file 3: Table S2). For example, in younger patients (commercially insured) with and without anemia, prevalence of arteriosclerotic heart disease (ASHD) was 23.6% vs. 13.9%, congestive heart failure (CHF) 21.4% vs. 9.0%, dysrhythmia 19.5% vs. 9.5%, and glomerulonephritis 7.8% vs. 3.8%. In older patients (Medicare) with and without anemia, corresponding proportions were 52.2% vs. 36.4%, 40.5% vs. 20.1%, 43.8% vs. 28.0%, and 6.0% vs. 2.7%, respectively. Prevalence of comorbid conditions also increased with increasing CKD stage (Additional file 4: Table S3).

### Anemia treatment patterns in stage 3–5 NDD-CKD patients with anemia

**Fig. 2:**
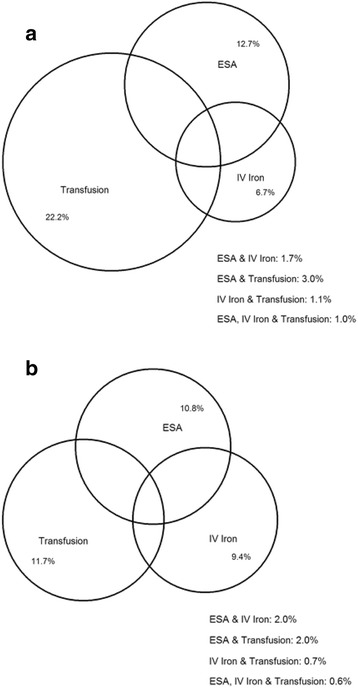
Proportion of stage 3–5 non-dialysis-dependent chronic kidney disease patients with anemia treated with erythropoietin-stimulating agents, intravenous iron and/or red blood cell transfusion. Panel (**a**), Medicare-covered patients aged 66–85 years; panel (**b**), Commercially insured patients aged 18–63 years. ESA, erythropoietin-stimulating agents; IV, intravenous

Of 15,716 younger patients with anemia identified from MarketScan data, 26.2% received at least one type of treatment (Fig. 2). Specifically, 11.7%, 10.8%, and 9.4% were treated with RBC transfusion, ESAs, and IV iron, respectively (Additional file 5: Table S4). For those receiving treatment, median times from anemia diagnosis to treatment were 21 (interquartile range [IQR] 97), 33 (IQR 117), and 44 (IQR 139) days for ESAs, RBC transfusion, and IV iron, respectively. Among all treated patients, 20% received two or more treatments (e.g., RBC transfusion and ESA). Treatment patterns were similar among Medicare patients, with larger proportions receiving treatment. Of 109,251 older patients with anemia, 34.0% received at least one type of treatment for anemia. Specifically, 22.2%, 12.7%, and 6.7% were treated with RBC transfusion, ESAs, and IV iron, respectively. For those receiving treatment, median times from anemia diagnosis to treatment were 18 (IQR 92), 34 (IQR 150), and 84 (IQR 169) days for ESAs, RBC transfusion, and IV iron, respectively.

While 10.8% of commercially insured patients and 12.7% of Medicare patients received ESA treatment, less than 5% received treatment consistently (defined as ESA administration in 80% of available months).

### Healthcare utilization in stage 3–5 NDD-CKD patients with and without anemia

**Fig. 3:**
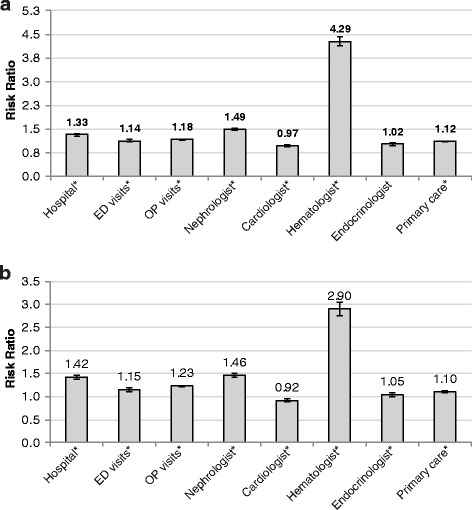
Adjusted hazard ratios of healthcare utilization in stage 3–5 non-dialysis dependent chronic kidney disease patients with and without anemia. Panel (**a**), Medicare-covered patients aged 66–85 years; panel (**b**), commercially insured patients aged 18–63 years. Results were from Poisson regression models with number of each type of health service as the dependent variable adjusting for patient demographics, baseline comorbidities, inflammatory conditions, and CKD stage. CKD, chronic kidney disease; ED, emergency department; OP, outpatient. ^*^P < 0.05

Unadjusted and adjusted healthcare resource utilization in patients with and without anemia is presented in Tables 2 and 3. In general, anemia was associated with greater resource use. After adjusting for patient case-mix, hospital admissions for anemic patients were 1.33 (95% confidence interval [CI], 1.31–1.34) and 1.42 (95% CI, 1.38–1.47) times higher for older and younger patients, respectively, and hematologist visits 4.29 (95% CI, 4.08–4.52) and 2.90 (95% CI, 2.76–3.04) times higher, respectively (Fig. 3).

**Table 2:** Healthcare utilization in stage 3–5 NDD-CKD Patients, Medicare and commercially insured (MarketScan) datasets

	Medicare^a^			Commercially insured		
	Unadjusted		Adjusted^b^	Unadjusted		Adjusted^a^
Healthcare utilization	Non-anemia	Anemia	Relative risk (95% CI)	Non-anemia	Anemia	Relative risk (95% CI)
Number of claims, PPY						
Hospitalizations	0.45	0.93	1.33 (1.31–1.34)	0.25	0.63	1.42 (1.38–1.47)
ED visits	0.50	0.75	1.14 (1.12–1.15)	0.34	0.58	1.15 (1.11–1.19)
SNF admissions	0.21	0.55	1.56 (1.53–1.59)	–	–	–
Hospice admissions	0.10	0.27	1.52 (1.48–1.55)	–	–	–
Outpatient visits	23.77	31.74	1.18 (1.18–1.18)	20.59	32.01	1.23 (1.22–1.23)
Length of hospitalization for all patients (days)						
Mean (SD)	2.36 (7.47)	5.24 (12.39)	–	0.93 (5.03)	2.61 (10.38)	–
Median	0.00	0.00	–	0.00	0.00	–
IQR	1.00	5.25		0.00	0.00	
Length of hospitalization for hospitalized patients (days)						
Mean (SD)	9.14 (12.42)	12.49 (16.60)	–	7.52 (12.47)	10.96 (19.01)	–
Median	5.00	7.00	–	4.00	5.00	–
IQR	8.00	12.00		6.00	9.00	
Physician visits, PPY						
Nephrologist	1.21	1.97	1.49 (1.48–1.50)	1.02	1.60	1.46 (1.43–1.50)
Cardiologist	2.40	3.06	0.97 (0.96–0.97)	0.69	0.94	0.92 (0.90–0.95)
Hematologist	0.02	0.09	4.29 (4.08–4.52)	0.11	0.46	2.90 (2.76–3.04)
Endocrinologist	0.35	0.39	1.02 (1.00–1.03)	0.27	0.33	1.05 (1.00–1.09)
Primary care practitioner	7.75	9.86	1.12 (1.11–1.12)	4.03	5.27	1.10 (1.09–1.12)

*CKD* chronic kidney disease, *NDD* non-dialysis-dependent, *ED* emergency department, *PPY* per person-year, *SNF* skilled nursing facility

^a^Including patients aged 66–85 years

^b^Poisson regressions adjusted for demographics, comorbid conditions, inflammatory conditions, and CKD stage

**Table 3:** Unadjusted healthcare utilization in stage 3–5 NDD-CKD Patients by stage, medicare and commercially insured (MarketScan) datasets

	Medicare^a^						Commercially insured					
	Anemia, CKD stage			Non-anemia, CKD stage			Anemia, CKD stage			Non-anemia, CKD stage		
Healthcare utilization	3	4	5	3	4	5	3	4	5	3	4	5
Number of claims, PPY												
Hospitalizations	0.83	1.03	1.30	0.41	0.60	0.68	0.56	0.65	0.90	0.22	0.35	0.49
ED visits	0.72	0.78	0.85	0.48	0.56	0.61	0.58	0.52	0.66	0.32	0.39	0.48
SNF admissions	0.50	0.59	0.78	0.19	0.30	0.36	–	–	–	–	–	–
Hospice admissions	0.24	0.29	0.41	0.09	0.14	0.25	–	–	–	–	–	–
Outpatient visits	30.87	33.09	33.99	23.48	25.01	25.26	31.28	32.46	34.34	20.08	22.43	24.24
Length of hospitalization, all patients (days)												
Mean	4.66	5.75	7.46	2.13	3.30	3.67	2.48	2.52	3.22	0.82	1.20	1.89
Median	0.00	0.00	1.00	0.00	0.00	0.00	0.00	0.00	0.00	0.00	0.00	0.00
Length of hospitalization, hospitalized patients (days)												
Mean	11.92	12.68	14.70	8.76	10.33	10.58	11.39	10.25	10.64	7.52	7.00	8.50
Median	7.00	8.00	8.00	5.00	6.00	6.00	5.00	5.00	4.00	4.00	4.00	3.00
Physician visits, PPY												
Nephrologist	1.31	3.00	3.63	1.05	2.01	1.82	1.16	2.31	2.39	0.88	1.70	1.42
Cardiologist	3.04	3.15	2.98	2.36	2.56	2.55	0.94	0.98	0.88	0.68	0.78	0.63
Hematologist	0.09	0.10	0.09	0.02	0.02	0.01	0.46	0.46	0.44	0.11	0.13	0.13
Endocrinologist	0.38	0.41	0.34	0.35	0.37	0.31	0.33	0.31	0.32	0.27	0.27	0.22
PCP	9.78	9.98	10.04	7.71	7.99	7.81	5.24	5.39	5.19	4.01	4.13	4.10

*CKD* chronic kidney disease, *ED* emergency department, *NDD* non-dialysis-dependent, *PCP* primary care physician/practitioner, *PPY* per patient-year, *SNF* skilled nursing facility

^a^Including patients aged 66–85 years

## Discussion

Our study greatly expands existing information on anemia prevalence in older Medicare-covered and younger commercially insured stage 3–5 NDD-CKD patients. Using two large datasets (Medicare and MarketScan), we showed that anemia prevalence was much higher (52%) in older than in younger (28%) patients. Anemia prevalence rose sharply with increasing CKD stage, from 22.4% in younger commercially insured stage 3 NDD-CKD patients to 53.9% in stage 5, and from 43.9% in older Medicare stage 3 patients to 72.8% in stage 5. Anemia prevalence was generally higher among women at each CKD stage. The most common anemia-related treatment across both data sets was RBC transfusion, followed by ESAs and IV iron. Only younger stage 5 NDD-CKD patients with anemia were more likely to be treated with ESAs than with RBC transfusion. After adjustment for patient case-mix, hospital admissions, nephrologist visits, and hematologist visits were significantly more frequent for anemic than for non-anemic patients.

Prior to our study, the only recent analysis of anemia prevalence in NDD-CKD patients was derived from NHANES 2007–2008 and 2009–2010 datasets [2]. Prevalence of CKD and anemia were defined by laboratory values (hemoglobin levels < 12 g/dL in adult women and < 13 g/dL in adult men), which is ideal; however, only 410 patients with CKD in the dataset could be evaluated, so subgroup analysis was not possible. This analysis showed that 15.4% of CKD patients (stages 1–5, including dialysis) had anemia. The authors found a weighted anemia prevalence of 17.4% (*n* = 231), 50.3% (*n* = 37), and 53.4% (*n* = 17) for CKD stage 3, 4, and 5 (likely including dialysis patients), respectively. In contrast, our evaluation included 109,251 older Medicare-covered and 15,716 younger commercially insured patients with CKD and anemia. We found CKD stage-specific anemia prevalence similar to NHANES findings in the younger (aged ≤63 years) commercially insured population, 22.4%, 41.3%, and 53.9% for CKD stages 3, 4, and 5 (not including dialysis patients), respectively, but higher anemia prevalence in the older Medicare-covered population, 46.2%, 65.8%, and 73.8% for CKD stages 3, 4, and 5 (not including dialysis patients), respectively. The age distribution was not included in the NHANES analysis, only the information that participants were adults aged older than18 years [2], so direct age-specific comparison to our data is not possible.

Anemia prevalence in stage 3–5 NDD-CKD patients increased monotonically as age increased, similar to the general population. Using NHANES data from 2003 to 2012, Le et al. showed that weighted anemia prevalence in the US increased by age from 3.8% for ages 15–29 years to 19.4% for ages 80–85 years [13]. Not surprisingly, and in contrast to Le’s findings, we found anemia prevalence several-fold higher in NDD-CKD patients than in the general population; prevalence was 25.9% and 54.7%, respectively, among patients aged 18–44 and 80–84 years. Variability of anemia prevalence by sex was much less (Medicare: 53.2%, women; 46.9%, men; commercially insured: 33.6%, women; 23.7% men) than in the general population, where women are more than twice as likely as men to have anemia [13]. We also found less racial variability among NDD-CKD patients with anemia (Medicare only: black 57.9%, white 48.9%, other 51.4%) than in the general population, where non-Hispanic blacks were 2.4–3.7 times more likely to have anemia than other race groups [13].

In our updated analyses (2011–2013), we showed that both younger and older anemic patients were most likely to be treated with RBC transfusion (22.2%, older; 11.7%, younger), followed by ESAs (12.7%, older; 10.8%, younger), and IV iron (6.7%, older; 9.4%, younger). Two earlier studies showed lower use of RBC transfusions than we found. Lawler et al. identified anemia treatment in a predominantly older male Veterans Administration CKD population from 2003 to 2005 following a first hemoglobin level under 11 g/dL. Compared with our data, ESA use was lower (about 7%), and RBC transfusion use was lower (16.5%) than in the older Medicare population we evaluated [14]. These data are not surprising, as many Veterans Administration patients had not been diagnosed with anemia despite lower hemoglobin levels. We looked for anemia treatment after a documented anemia diagnosis. Lawler also showed that iron (oral and IV) use was 30.9%, which is not comparable to our data as we could track only IV iron use. Fox et al. evaluated transfusion burden in 374 NDD-CKD adult patients with severe anemia (hemoglobin < 10 g/dL) and a mean age of 71 years in the Henry Ford Health System from 2004 to 2008 [15]. They found that RBC transfusions were administered in 20% of these patients during a mean follow-up period of 459 days. We found a relatively higher rate of transfusions (22% over 1 year) in the older Medicare population. Several ESA trials showing increased risk of death, cardiovascular events, and stroke in NDD-CKD patients treated to higher hemoglobin targets of 13–15 g/dL [4–6], additional FDA boxed warnings for ESAs released in June 2011 [7], and KDIGO anemia guidelines released in 2012 [8] may have led to decreased ESA use in our study timeframe and affected anemia treatment selection.

Stauffer and Fan evaluated self-reported anemia treatment in NHANES 2007–2008 and 2009–2010 participants with CKD [2]. Of 410 CKD patients, 22.8% self-reported anemia treatment in the past 3 months (20.7%, 43.0%, and 22.8% for stage 3, 4, and 5, respectively), but specific treatments were not described. Winkelmayer et al. studied trends in anemia treatment, 1995–2010, among NDD-CKD patients aged 67 years or older 2 years before ESRD, using US Renal Data System (USRDS) data. They showed that ESA use peaked in 2007 at 40.8%, but declined to 35.0% in 2010, while IV iron and RBC transfusions were used in 12.3% and 40.3%, respectively, in 2010 [16]. Their data most closely compare to our data for stage 5 NDD-CKD patients; in a contemporary Medicare cohort (2011–2013), we showed lower rates with 21.8% receiving ESAs, 9.6% IV iron, and 31.2% RBC transfusions. Lower use of all treatments in 2012 may be attributable to less severe anemia in our NDD-CKD stage 5 patients than in Winkelmayer’s cohort, all of whom initiated dialysis. Lower ESA use by 2012 may also be due to additional FDA boxed warnings added to ESA package inserts in June 2011 [7].

Thamer et al. evaluated ESA use among stage 3 and 4 NDD-CKD patients before and after the TREAT study (2007–2011) using the MarketScan database [3]. In patients aged younger than 65 years, they showed that 7.6% and 22.9% with CKD stages 3 and 4, respectively, were treated with ESAs from September 2009 to October 2011; in a more contemporary similarly aged younger population, we show even lower ESA use (6.5% and 15.7%), suggesting that FDA action in June 2011 [7] may have further affected ESA use. We did not evaluate patients aged older than 65 years using MarketScan data because of potential missing data for patients with both commercial and Medicare insurances.

In our 2012 cohort of stage 3–5 NDD-CKD patients, RBC transfusions were used more than ESAs following anemia diagnosis; 22.2% of older Medicare and 11.7% of younger commercially insured patients had at least one RBC transfusion after anemia diagnosis (within means of 2.7 and 2.4 months, respectively). Only younger anemic patients were more likely to receive an ESA (19.9%) than an RBC transfusion (15.0%) after anemia diagnosis. RBC transfusions as a common treatment following anemia diagnosis in NDD-CKD patients is concerning. Leffell et al. merged serial HLA antibody data with USRDS data for 1324 patients on the kidney transplant waiting list, 2004–2010. Using a matched patient cohort and a cross-over cohort, they showed that 20% of patients who received leukodepleted RBC transfusions, compared with 3% who did not, displayed an antibody response (*P* = 0.001). The response was similar in the crossover cohort [17]. Notably, they showed increased panel-reactive antibody values in 26.3% of transfusion patients compared with 5.8% of non-transfusion patients. Yabu et al. showed similar results in a patient population with a smaller percentage of black patients [18]. The mean waiting time for a kidney transplant was 2 months longer, on average, for transfused than for non-transfused patients [19]. Thus, our finding that RBC transfusions are common in both older and younger stage 4 and 5 NDD-CKD patients has potential implications regarding antibody sensitization and increased waiting times if these patients meet requirements for future kidney transplant.

We found that that healthcare utilization was higher for stage 3–5 NDD-CKD patients with than without anemia. Other studies have identified anemia in NDD-CKD patients as a risk factor for increased hospitalizations and hospital days [9], although this relationship may be confounded by common underlying comorbid conditions such as CHF with fluid retention [20]. Garlo et al. found that stage 3–5 NDD-CKD patients (controlling for CHF) with hemoglobin levels 9 g/dL or lower compared with above 9 g/dL admitted to tertiary referral hospital had increased lengths of stay, but no difference in 30-day readmission rates [10]. We showed that after adjustment for multiple baseline comorbid conditions, including CHF, younger and older NDD-CKD patients with diagnosed anemia had significantly more hospitalizations, ED visits, and SNF admissions, along with increased hospital lengths of stay, than those without anemia. Outpatient visits, and specifically visits to nephrologists, hematologists, and primary care practitioners were also significantly higher in patients with diagnosed anemia. A small prospective observational study of nursing home residents with eGFR below 60 mL/min/1.73 m^2^ observed that residents with anemia showed greater earlier decline in a mobility distance measure, but not in other short-term or longer-term performance or self-report measures, than those without anemia [21]. CKD-related anemia affects many body systems but in particular the cardiovascular system. Anemia contributes to increased cardiac output, development of left ventricular hypertrophy and chronic heart failure; thus, it is not unexpected that healthcare utilization is higher among CKD patients with than without anemia.

Interestingly, we showed that anemia in CKD was associated with slightly fewer cardiologist visits, which seems counterintuitive given higher rates of ASHD and CHF in CKD patients with anemia versus without. A recent analysis comparing incident hemodialysis patients pre- and post-changes in CMS reimbursement policy and FDA label action showed that the risks of major adverse cardiovascular events (MACE), death, hospitalization for heart failure, and venous thromboembolism were similar in the pre- and post-policy periods, with lower stroke and myocardial infarction risk despite lower ESA use and higher rates of RBC transfusions; black patients showed significant reductions in risks of MACE and death [22]. Whether the same effect will occur in NDD-CKD patients or patient subgroups, or whether treatment of NDD-CKD anemia to lower hemoglobin target levels with ESAs, or alternative therapies such as hypoxia-inducible factor (HIF) prolyl hydroxylase inhibitors, will reduce mortality, particular cardiovascular outcomes, or overall healthcare utilization is unclear.

Our study has several strengths. We used two large national datasets to evaluate anemia prevalence, treatment, and healthcare utilization in older Medicare-covered and younger commercially insured patients with stages 3–5 CKD. We conducted several subgroup analyses to evaluate anemia prevalence by CKD stage, age, and race, which were not possible using the NHANES dataset. We also considered more contemporary trends in anemia treatment and included ESAs, IV iron, and RBC transfusion use, as previous studies did not. We compared broad and specific key categories in inpatient and outpatient healthcare utilization for NDD-CKD patients with and without anemia, adjusted for multiple factors.

Our study also has several limitations. This is a retrospective analysis of anemia using claims-based data that are not collected for research purposes, so regression relationships should be considered associations and not causal. Although we used multiple covariates as adjusters in our models, residual confounding may be present. Hemoglobin values were not available for NDD-CKD patients in the Medicare dataset, and were available for less than 5% of MarketScan patients, so we could not confirm anemia diagnosis with information on anemia severity. We evaluated anemia prevalence across age categories using two different datasets (Medicare for older, MarketScan for younger patients); data from these databases are not directly comparable as demographic and comorbidity characteristics differ among the populations. We chose not to evaluate anemia prevalence and treatment in older MarketScan individuals because previous research showed that 100% of claims may not be identified for patients with both commercial and Medicare insurances. Since the anemia of CKD is often a diagnosis of exclusion and can be seen in combination with other anemia types, we are not certain that all of the anemia diagnoses we identified were CKD-related. However, we used a timeframe around the CKD index date to increase the probability that anemia was related to CKD. We did not assess oral iron treatment, as most oral iron therapy is available over the counter and use is not reliably identified in Medicare or commercial prescription claims databases. Oral iron therapy is likely to be the predominant iron therapy for NDD-CKD patients, compared with predominance of IV iron for hemodialysis patients. In addition to general lack of laboratory information, we did not have information on fluid status, which may confound the relationship between CKD anemia and outcomes.

## Conclusions

Our study is the largest to date examining anemia prevalence in stage 3–5 NDD-CKD older Medicare-covered and younger commercially insured patients, allowing analysis by important subgroups such as age, race (Medicare only), and CKD-stage. In a 2012 patient cohort, both younger and older patients with stage 3–5 NDD-CKD and diagnosed anemia were more likely to be treated with an RBC transfusion than with ESAs or IV iron. This is concerning due to the potential for increased panel-reactive antibodies and increased waiting time for kidney transplant. Although we showed increased healthcare utilization for NDD-CKD patients with anemia versus those without, further research is needed to determine whether future therapies such as HIF prolyl hydroxylase inhibitors, anemia treatment to lower hemoglobin targets with ESAs and iron, or treatment of specific subgroups (e.g., CKD with heart failure) can improve health outcomes and reduce healthcare utilization.

## Additional files

Additional file 1: **Table S1.** Codes Used to Identify CKD, Anemia, Treatment, and Comorbid Conditions. (PDF 35 kb)

Additional file 2: **Figure S1.** Flowchart for patient selection. CKD, chronic kidney disease; ESRD, end-stage renal disease; HMO, health maintenance organization. (JPEG 252 kb)

Additional file 3: **Table S2.** Baseline Characteristics and Comorbid Conditions in Stage 3–5 NDD-CKD Patients, 20% Medicare and Commercially Insured (MarketScan) Datasets. (PDF 105 kb)

Additional file 4: **Table S3.** Baseline Characteristics and Comorbidity in Stage 3–5 NDD-CKD Patients by Stage, 20% Medicare and Commercially Insured (MarketScan) Datasets. (PDF 91 kb)

Additional file 5: **Table S4.** Anemia Treatment in Stage 3–5 NDD-CKD Patients with Anemia by Stage, 20% Medicare and Commercially Insured (MarketScan) Datasets. (PDF 70 kb)

## Abbreviations

ASHD: Atherosclerotic heart disease
CHF: Congestive heart failure
CKD: Chronic kidney disease
CMS: Centers for Medicare & Medicaid Services
ED: Emergency department
eGFR: Estimated glomerular filtration rate
ESA: Erythropoiesis-stimulating agent
ESRD: end-stage renal disease
FDA: Food and Drug Administration
HIF: Hypoxia-inducible factor
ICD-9-CM: International Classification of Diseases, Ninth Revision, Clinical Modificaiton
IQR: Interquartile range
IV: Intravenous
KDIGO: Kidney Disease Improving Global Ourcomes
MACE: Major adverse cardiovascular evennts
NDC: National drug code
NDD: Non-dialysis-dependent
NHANES: National Health and Nutrition Examination Survey
RBC: Red blood cell
SAF: Standard analysis file
SNF: Skilled nursing facility
TREAT: Trial to Reduce Cardiovascular Events with Aranesp Therapy
USRDS: United States Renal Data System
