# Supplementary material for: Prevalence, treatment patterns, and healthcare resource utilization in Medicare and commercially insured non-dialysis-dependent chronic kidney disease patients with and without anemia in the United States
Source: BMC Nephrol. 2018 Mar 15;19:67. doi: 10.1186/s12882-018-0861-1 (PMC5856223; doi:10.1186/s12882-018-0861-1)
Supplement: Supplementary file 1 — Table S1. Codes Used to Identify CKD, Anemia, Treatment, and Comorbid Conditions. (PDF 35 kb) [file 12882_2018_861_MOESM1_ESM.pdf]

Table S1. Codes Used to Identify CKD, Anemia, Treatment, and Comorbid Conditions

Disease or Condition	Codes
CKD	ICD-9-CM diagnosis codes: 016.0; 095.4; 189.0; 189.9; 223.0; 236.91; 250.4; 271.4; 274.1; 283.11; 403.X0; 403.X1; 404.X0; 404.X1; 404.X2; 404.X3; 440.1; 442.1; 447.3; 572.4; 580-588; 591; 642.1; 646.2; 753.12-753.17; 753.19; 753.2; 794.4.
Anemia	ICD-9-CM diagnosis codes: 280.0; 280.1; 280.8; 280.9; 281.0; 281.1; 281.2; 281.3; 281.4; 281.8; 281.9; 285.1; 285.21; 285.29; 285.8; 285.9.
Comorbid conditions	ICD-9-CM diagnosis codes or ICD-9-CM V codes
ASHD	410-414; V45.81; V45.82
CHF	398.91;422; 425; 428; 402.X1; 404.x1; 404.x3; V42.1
CVA/TIA	430-438
PVD	440-444; 447; 451-453; 557
Cardiac (other)	420-421;423-424; 429; 785.0-785.3; V42.2;V43.3
COPD	491-494; 496; 510
GI	456.0-456.2; 530.7; 531-534; 569.84; 569.85; 578
Liver disease	570; 571; 572.1; 572.4; 573.1-573.3; V42.7
Dysrhythmia	426-427; V45.0; V53.3
Cancer	140-172; 174-208; 230-231; 233-234
Diabetes	250; 357.2; 362.0x; 366.41
Hypertension	362.11; 401.x-405.x; 437.2
Inflammatory conditions	ICD-9-CM diagnosis codes or ICD-9-CM V codes
Chronic infections	730.1; 424.90; 042; 010-018; 117.5; 114; 117.3; 136.3
Crohn’s disease	555
Ulcerative colitis	556
Hepatitis C	070.41, 070.44, 070.51, 070.54, 070.70, 070.71
Gout	274; V77.5
Rheumatoid arthritis	714
Anemia treatment	
ESAs: HCPCS Codes	EPO: HCPCS: ‘J0885’, ‘J0886’, ‘Q4081’, ‘Q0136’, ‘Q4055’; and revenue code 0634 and 0635<br>DPO: HCPCS: 'C1774', 'J0880', 'Q4054', 'Q0137', 'J0881', 'J0882'<br>PEG: HCPCS: 'Q2047', 'J0890'
ESAs: NDC Codes	420230260', '548682523', '548685673', '555130126', '555130144', '555130148', '555130267', '555130283', '555130478', '555130823', '596760302', '596760303', '596760304', '596760310', '596760312', '596760320', '596760340', '635520478', '635520823', '54569313700', '54868580200', '246460090', '246460091', '246460092', '246460093', '246460094', '246460095', '246460096', '246460097', '548685429', '548685867', '555130002', '555130003', '555130004', '555130005', '555130006', '555130008', '555130010', '555130011', '555130012', '555130013', '555130013', '555130015', '555130021', '555130023', '555130025', '555130027', '555130028', '555130032', '555130039', '555130041', '555130043', '555130044', '555130046', '555130048', '555130053', '555130054', '555130057', '555130090', '555130091', '555130092', '555130093', '555130094', '555130095', '555130096', '555130097', '555130110', '555130111', '597030043', '55513005804', '55513005801', '55513003704', '55513003701', '54868542800', '00062030302', '00062030402', '00062031002', '00062740003', '00062740103', '00062740201', '00062740501', '55513001401', '55513001404', '64764061010', '64764062020'
Intravenous iron	HCPCS codes: J1760 - J1780; J2915; J2916; J1750; J1751; J1752; J1755; J1756; J1760; J1770; J1780; J1442; J1443; W0091; W0121; W0231; W0233; X0011; X0107; Q0138; Q0139

RBC transfusion	Revenue codes: 0380; 0381; 0382; 0391; value code: 37; procedure codes: 99.03; 99.04; IP/SNF/OP/Medicare Part B HCPCS/CPT: P9010, P9011, P9016, P9021, P9022, P9038, P9039, P9040, P9051, P9054, P9056, P9057, P9058, 36430

ASHD, atherosclerotic heart disease; CHF, congestive/chronic heart failure; CKD, chronic kidney disease; COPD, chronic obstructive pulmonary disease; CPT, Current Procedural Terminology; CVA/TIA, cerebrovascular accident/transient ischemic attack; DPO, darbepoietin; EPO, erythropoietin; ESAs: erythropoietin-stimulating agents; GI, gastrointestinal; HCPCS, Healthcare Common Procedure Coding System; IP, inpatient; ICD-9-CM, International Classification of Diseases, Ninth Revision, Clinical Modification; NDC, National Drug Code; OP, outpatient; PEG, peginesatide (*note:* withdrawn from US market in February 2013); PVD, peripheral vascular disease; RBC, red blood cell; SNF, skilled nursing facility.
