# Supplementary figures and images for: Prevalence, treatment patterns, and healthcare resource utilization in Medicare and commercially insured non-dialysis-dependent chronic kidney disease patients with and without anemia in the United States
Source: BMC Nephrol. 2018 Mar 15;19:67. doi: 10.1186/s12882-018-0861-1 (PMC5856223; doi:10.1186/s12882-018-0861-1)

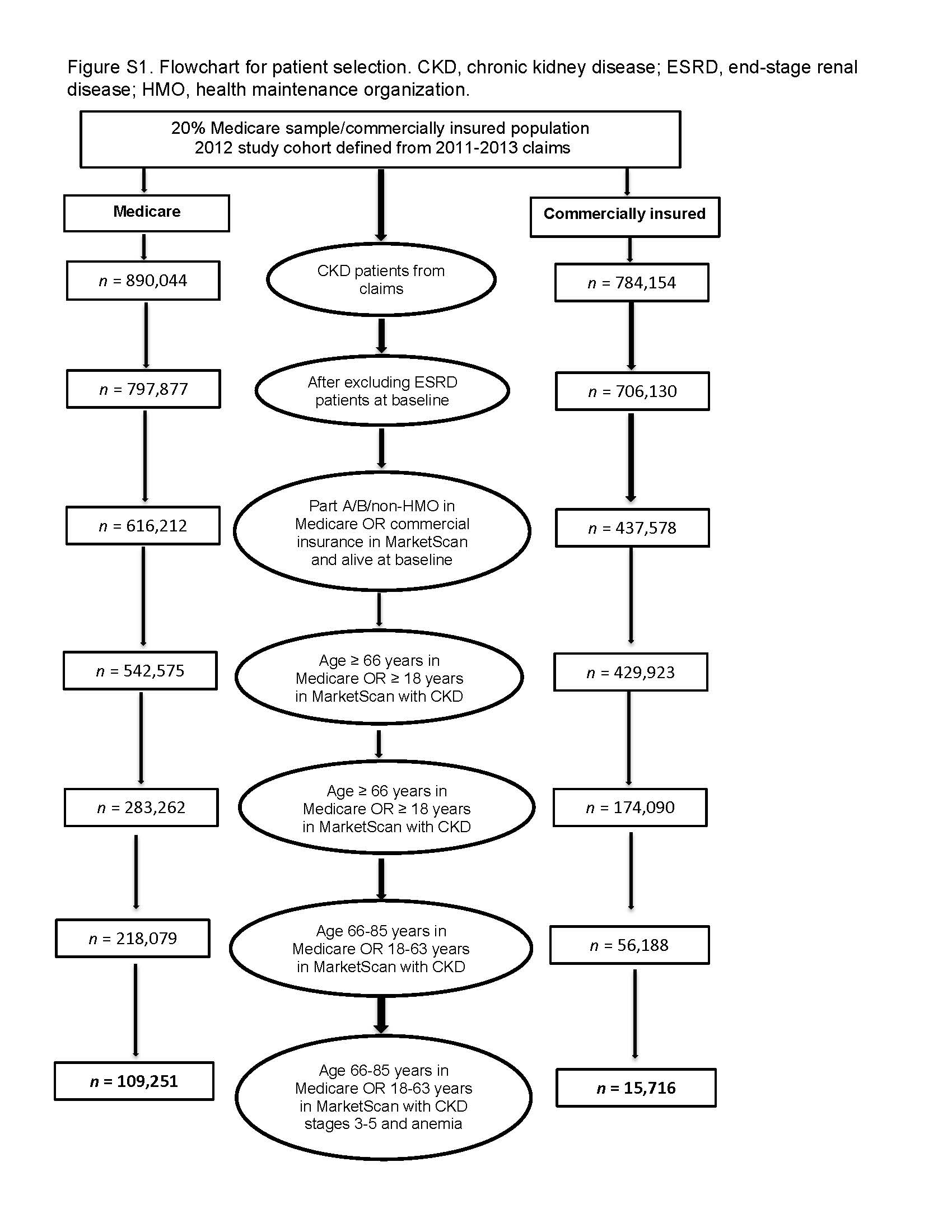

Supplement: Supplementary file 2 — Figure S1. Flowchart for patient selection. CKD, chronic kidney disease; ESRD, end-stage renal disease; HMO, health maintenance organization. (JPEG 252 kb) [file 12882_2018_861_MOESM2_ESM.jpg]
