# Supplementary material for: Prevalence, treatment patterns, and healthcare resource utilization in Medicare and commercially insured non-dialysis-dependent chronic kidney disease patients with and without anemia in the United States
Source: BMC Nephrol. 2018 Mar 15;19:67. doi: 10.1186/s12882-018-0861-1 (PMC5856223; doi:10.1186/s12882-018-0861-1)
Supplement: Supplementary file 3 — Table S2. Baseline Characteristics and Comorbid Conditions in Stage 3–5 NDD-CKD Patients, 20% Medicare and Commercially Insured (MarketScan) Datasets. (PDF 105 kb) [file 12882_2018_861_MOESM3_ESM.pdf]

Table S2. Baseline Characteristics and Comorbid Conditions in Stage 3-5 NDD-CKD Patients, 20% Medicare and Commercially Insured (MarketScan) Datasets

Characteristics	Medicare			Commercially Insured		
	Anemia	No Anemia	Standardized Difference*	Anemia	No Anemia	Standardized Difference*
n of patients	109,251	108,828	——	15,716	40,472	——
Age in years						
18-44	——	——	——	11.0	12.2	3.9
45-54	——	——	——	23.9	25.1	2.7
55-59	——	——	——	28.3	27.6	-1.5
60-63	——	——	——	36.8	35.1	-3.6
66-69	14.2	17.7	9.6	——	——	——
70-74	23.1	26.1	7.0	——	——	——
75-79	27.1	27.1	0.0	——	——	——
80-85	35.6	29.1	-13.9	——	——	——
Sex						
Men	46.3	52.6	12.6	47.9	60.0	24.5
Women	53.7	47.4	-12.6	52.1	40.0	-24.5
Race†						
White	80.2	84.3	10.7	——	——	——
Black	14.0	10.2	-11.7	——	——	——
Other	5.8	5.5	-1.3	——	——	——
Comorbid conditions						
ASHD	52.2	36.4	-32.2	23.6	13.9	-24.9
CHF	40.5	20.1	-45.5	21.4	9.0	-35.0
CVA/TIA	23.6	14.2	-24.2	10.0	4.8	-20.1
PVD	35.6	21.1	-32.6	19.1	7.5	-34.5
Cardiac (other)	35.5	18.7	-38.5	23.5	9.7	-37.7
COPD	33.8	20.5	-30.2	15.7	7.9	-24.2
GI	12.6	1.9	-42.2	8.9	1.4	-34.7
Liver disease	3.7	1.5	-13.9	7.1	2.5	-21.5
Dysrhythmia	43.8	28.0	-33.4	19.5	9.5	-28.7
Cancer	20.6	13.6	-18.7	14.2	7.7	-20.9

Diabetes	58.7	50.3	-16.9	52.5	40.2	-24.9
Hypertension	96.5	92.9	-16.1	85.4	73.8	-29.1
Inflammatory conditions						
Glomerulonephritis	6.0	2.7	-16.2	7.8	3.8	-17.0
Chronic infections	1.9	0.7	-10.6	4.0	1.2	-17.5
Crohn’s disease	0.8	0.3	-6.8	1.3	0.5	-8.4
Ulcerative colitis	0.8	0.3	-6.8	1.1	0.4	-8.9
Hepatitis C	0.6	0.3	-4.5	2.3	0.9	-10.5
Gout	16.3	11.4	-14.2	9.7	7.9	-6.2
Rheumatoid arthritis	5.1	2.5	-13.6	3.1	1.8	-8.3

ASHD, atherosclerotic heart disease; CHF, congestive/chronic heart failure; CKD, chronic kidney disease; COPD, chronic obstructive pulmonary disease; CVA/TIA, cerebrovascular accident/transient ischemic attack; GI, gastrointestinal; NDD, non-dialysis-dependent; PVD, peripheral vascular disease.

*Standardized (mean) difference is a measure of distance between two group means. Standardized values are not affected by sample size; thus, comparison across variables on different scales is possible. Differences were considered significant if the absolute value of the standardized difference was greater than 10.

†Race variable is not available in the MarketScan database.
