# Supplementary material for: Prevalence, treatment patterns, and healthcare resource utilization in Medicare and commercially insured non-dialysis-dependent chronic kidney disease patients with and without anemia in the United States
Source: BMC Nephrol. 2018 Mar 15;19:67. doi: 10.1186/s12882-018-0861-1 (PMC5856223; doi:10.1186/s12882-018-0861-1)
Supplement: Supplementary file 4 — Table S3. Baseline Characteristics and Comorbidity in Stage 3–5 NDD-CKD Patients by Stage, 20% Medicare and Commercially Insured (MarketScan) Datasets. (PDF 91 kb) [file 12882_2018_861_MOESM4_ESM.pdf]

Table S3. Baseline Characteristics and Comorbidity in Stage 3-5 NDD-CKD Patients by Stage, 20% Medicare and Commercially Insured (MarketScan) Datasets

	Medicare						Commercially Insured					
	Anemia, CKD Stage			Non-Anemia, CKD Stage			Anemia, CKD Stage			Non-Anemia, CKD Stage		
Characteristics	3	4	5	3	4	5	3	4	5	3	4	5
n of patients	69,209	28,547	11,495	88,502	16,023	4,303	9,519	3,713	2,484	33,068	5,281	2,123
Age, years												
18-44	——	——	——	——	——	——	9.8	10.7	15.9	11.5	14.0	18.3
45-54	——	——	——	——	——	——	23.4	24.5	25.0	24.7	25.8	28.5
55-59	——	——	——	——	——	——	29.0	26.7	28.2	27.9	26.5	25.6
60-63	——	——	——	——	——	——	37.9	38.1	30.9	35.8	33.8	27.6
66-69	14.0	13.0	18.1	17.9	15.5	21.7	——	——	——	——	——	——
70-74	23.3	22.3	23.8	26.5	23.9	26.1	——	——	——	——	——	——
75-79	27.3	26.9	26.4	27.0	27.4	26.3	——	——	——	——	——	——
80-84	35.4	37.8	31.7	28.5	33.2	26.0	——	——	——	——	——	——
Sex												
Men	46.7	44.2	49.1	52.4	52.0	57.6	47.4	46.0	52.8	60.1	59.7	60.4
Women	53.3	55.8	50.9	47.6	48.0	42.4	52.6	54.0	47.2	39.9	40.3	39.6
Race*												
White	81.6	79.9	72.8	84.7	84.1	75.9	——	——	——	——	——	——
Black	13.0	14.1	19.3	9.8	10.5	17.0	——	——	——	——	——	——
Other	5.4	6.0	7.9	5.5	5.4	7.0	——	——	——	——	——	——
Comorbid conditions												
ASHD	51.0	53.5	56.7	35.3	40.7	41.8	23.0	24.3	24.6	13.5	16.2	15.3
CHF	37.0	44.8	50.6	18.5	26.7	28.8	19.2	24.3	25.6	8.2	12.5	13.4
CVA/TIA	23.2	23.1	27.3	13.8	15.2	18.4	9.8	9.8	11.1	4.5	5.5	6.2
PVD	34.1	36.2	42.9	20.1	24.9	27.6	18.3	18.9	22.2	7.1	8.6	11.4
Cardiac (other)	34.6	35.6	41.0	18.3	19.9	22.8	22.7	22.8	27.4	9.2	10.4	15.1
COPD	33.2	33.7	37.6	19.9	22.1	25.8	16.4	13.5	16.3	7.8	8.0	9.3
GI bleeding	12.3	12.2	15.8	1.8	1.8	3.0	8.5	7.9	12.0	1.3	1.1	3.7
Liver disease	3.6	3.4	5.7	1.5	1.3	2.6	6.6	5.1	12.1	2.3	2.3	6.0
Dysrhythmia	42.9	44.0	48.3	27.2	31.5	32.7	19.0	18.9	22.2	9.2	10.3	12.4
Cancer	21.2	19.6	19.7	13.6	13.5	13.5	15.1	13.2	12.2	7.9	6.8	6.4
Diabetes	56.3	62.1	65.0	49.1	55.3	54.3	51.3	56.5	50.7	39.2	46.0	41.0

Hypertension	95.9	97.4	97.4	92.6	94.9	92.0	84.1	87.5	87.1	73.1	77.6	75.1
Inflammatory conditions												
Glomerulonephritis	4.3	8.4	10.3	2.4	4.2	3.7	6.4	9.6	10.6	3.5	6.0	4.1
Chronic infections	1.9	1.8	2.8	0.6	0.8	0.9	3.8	4.0	4.5	1.2	1.2	2.0
Crohn’s disease	0.7	0.8	0.9	0.3	0.3	0.3	1.4	1.1	1.1	0.5	0.6	0.7
Ulcerative colitis	0.9	0.7	0.9	0.4	0.3	0.3	1.2	0.8	1.2	0.4	0.4	0.3
Hepatitis C	0.6	0.6	1.3	0.3	0.4	1.0	2.1	1.6	3.9	0.8	1.1	2.7
Gout	14.1	20.2	20.3	10.7	15.0	12.9	9.3	10.7	9.5	7.7	9.7	7.5
Rheumatoid arthritis	5.3	4.8	4.9	2.6	2.3	1.9	3.7	2.7	1.7	1.9	1.5	1.6

ASHD, atherosclerotic heart disease; CHF, congestive heart failure; CKD, chronic kidney disease; COPD, chronic obstructive pulmonary disease; CVA/TIA, cerebrovascular accident/transient ischemic attack; GI, gastrointestinal; NDD, non-dialysis-dependent; PVD, peripheral vascular disease.

*Race variable is not available in the MarketScan database.
