# Supplementary material for: Prevalence, treatment patterns, and healthcare resource utilization in Medicare and commercially insured non-dialysis-dependent chronic kidney disease patients with and without anemia in the United States
Source: BMC Nephrol. 2018 Mar 15;19:67. doi: 10.1186/s12882-018-0861-1 (PMC5856223; doi:10.1186/s12882-018-0861-1)
Supplement: Supplementary file 5 — Table S4. Anemia Treatment in Stage 3–5 NDD-CKD Patients with Anemia by Stage, 20% Medicare and Commercially Insured (MarketScan) Datasets. (PDF 70 kb) [file 12882_2018_861_MOESM5_ESM.pdf]

Table S4. Anemia Treatment in Stage 3-5 NDD-CKD Patients with Anemia by Stage, 20% Medicare and Commercially Insured (MarketScan) Datasets

Treatment	Medicare,* CKD Stage				Commercially Insured, CKD Stage			
	3-5	3	4	5	3-5	3	4	5
Total *n*	109,251	69,209	28,547	11,495	15,716	9519	3713	2484
Patients treated, %								
ESA	12.7	8.8	18.5	21.8	10.8	6.5	15.7	19.9
IV iron	6.7	5.6	8.3	9.6	9.4	7.8	11.3	12.9
RBC transfusion	22.2	20.5	22.6	31.2	11.7	10.9	11.7	15.0
Any	34.0	29.4	39.1	48.8	26.2	21.3	31.1	37.4
Combination treatments, %†								
ESAs only	7.1	5.0	10.7	11.3	6.3	3.7	9.6	11.1
IV iron only	3.0	2.8	3.4	3.4	6.2	5.9	7.2	6.2
RBC transfusion only	17.2	16.8	16.1	22.3	8.5	8.4	7.3	10.7
ESAs and IV iron only	1.7	1.1	2.5	3.0	2.0	0.9	2.6	5.1
ESAs and RBC transfusion only	3.0	2.1	4.1	5.7	2.0	1.5	2.8	2.7
IV iron and RBC transfusion only	1.1	1.0	1.1	1.4	0.7	0.7	0.9	0.6
ESAs and IV iron and RBC transfusion	1.0	0.7	1.3	1.8	0.6	0.4	0.7	0.9

*Including patients aged 66-85 years.

†Groups are mutually exclusive.

CKD, chronic kidney disease; ESA, erythropoiesis-stimulating agent; IV, intravenous; NDD, non-dialysis-dependent; RBC, red blood cell.
